# Optimizing Endodontic Surgery: A Systematic Review of Guided Tissue Regeneration, Grafting, and Platelet Concentrates vs. No Intervention

**DOI:** 10.3390/dj13030091

**Published:** 2025-02-20

**Authors:** Mohammad Sabeti, Natalie Black, Mohsen Ramazani, Nafiseh Zarenejaddivkolahei, Mahmood Moosazadeh

**Affiliations:** 1 Advanced Specialty Program in Endodontics, UCSF School of Dentistry, 707 Parnassus Ave. Room-D 3226, San Francisco, CA 94143, USA; 2 UCSF Advanced Specialty Program in Endodontics, 707 Parnassus Ave. Room-D 3226, San Francisco, CA 94143, USA; natalie.black@ucsf.edu; 3 Iranian Center for Endodontic Research & Medical Education Research and Development, Mazandaran University of Medica Sciences, Sari 48175-866, Iran; m.ramezani@mazums.ac.ir; 4 Department of Restorative Dentistry and Endodontics, Dental School, Mazandaran University of Medica Sciences, Sari 48175-866, Iran; nzarenejad@mazums.ac.ir; 5 Noncommunicable Disease Institute, Mazandaran University of Medical Science, Sari 48175-866, Iran; mmoosazadeh1351@mazums.ac.ir

**Keywords:** periapical surgery, apicoectomy, endodontic surgery, bone graft, tissue regeneration, platelet concentrates, and membranes

## Abstract

**Background/Objectives:** Guided tissue regeneration (GTR) and the use of various grafting materials and platelet concentrates have emerged as promising adjunctive techniques in endodontic surgery to enhance bone regeneration and improve healing outcomes, although evidence regarding their consistent effectiveness remains inconclusive. The aim of this systematic review is to evaluate existing randomized controlled trials (RCTs) and prospective clinical trials to determine the efficacy of bone grafts, membranes, or platelet concentrates on outcomes in endodontic periapical surgery, employing a robust evidence-based approach. **Methods**: Searches were conducted in MEDLINE (PubMed), Embase, Cochrane Library, and gray literature databases from their inception until March 2024. Study selection and data extraction were conducted independently by two reviewers. Eligible randomized controlled trials (RCTs) and prospective clinical trials underwent critical appraisal for risk of bias and quality of evidence and were subjected to meta-analysis to determine treatment effects. **Results**: Twelve studies were included. The pool success rate for periapical surgery using any regenerative material (bone graft, membrane, or platelet concentrate) was 2.48 (OR: 2.48, 95% CI: 1.42–4.34). Multiple subgroup analyses based on the type of regenerative material used during treatment were performed, presenting high certainty of evidence. The subgroup analysis, which examined bone graft only, bone graft with membrane, membrane only, concentrated growth factor only, and concentrated growth factor with bone graft, yielded significant results only for concentrated growth factor with bone graft (OR: 15.01, 95% CI: 1.12–271.70). While the success rate of periapical surgery with other regenerative materials did not reach statistical significance, the effect size was substantial. **Conclusions:** Overall, the findings indicate that utilizing a concentrated growth factor with a bone graft significantly improves the success of bone regeneration procedures over a 12-month follow-up period compared to interventions without these components. However, more research will be needed with larger sample sizes and longer follow-up times.

## 1. Introduction

While primary endodontic therapy typically demonstrates a high success rate, there are instances where endodontic surgery becomes necessary for patients with persistent periradicular periodontitis that have not responded favorably to non-surgical endodontic approaches [1]. Endodontic surgery includes performing an osteotomy to access and excavate a periapical lesion while sealing off the root canal system from microorganisms. The success of post-operative outcomes following periapical surgery is intricately tied to the location and quantity of the remaining bone enveloping the root. In addressing the challenges presented by such scenarios, guided tissue regeneration (GTR) methods emerge as promising adjunctive strategies to enhance hard tissue formation during surgical endodontic procedures [2]. Periodontists rely on guided tissue regeneration (GTR) to stimulate bone formation. Research is underway to see if this technique can be beneficial in endodontic surgery as well. GTR employs a range of materials such as barrier membranes, bone grafts, and platelet concentrates to promote healing and tissue regrowth.

Presently, several materials are available for tissue and bone regeneration in endodontic periapical surgery. These include barrier membranes, bone replacement grafts, and host modulating agents such as platelet concentrates (APCs) [3]. Barrier membranes are typically categorized as non-resorbable or resorbable. Non-resorbable membranes such as ePTFE (Gore-Tex) necessitate a second surgical procedure for removal, increasing the risk of infection and patient complications due to site re-entry [4]. Various bone grafting techniques can be employed in endodontic surgery; however, autografts are currently considered the preferred approach. They are osteogenic and sourced from a different site within the same host, reducing the risk of disease transmission. However, accessing a second surgical site increases the risk of morbidity for patients [5]. Allografts, obtained from tissue banks, provide an alternative bone grafting material. They function mainly through osteoconduction, creating a framework for new bone to grow on. Additionally, they may exhibit some osteoinductive properties, attracting and stimulating bone-forming cells. Decalcified freeze-dried bone allograft and freeze-dried bone allograft are two examples of this type of graft material [6]. Another bone grafting option is xenografts, which are harvested from different animal species such as bovine or porcine. Similar to allografts, they primarily function through osteoconduction, providing a framework for new bone to form. However, due to potential immune reactions, xenografts require removal of most organic components before use [7]. Lastly, synthetic grafts, called alloplasts, are osteoconductive and come in variations like hydroxyapatite, non-ceramic polymer, bioactive glass, or beta-tricalcium phosphate. They act as space maintainers without regenerative potential [7,8]. Platelet concentrates (APCs), including platelet-rich plasma (PRP) and platelet-rich fibrin (PRF), have garnered significant attention for their healing properties in periapical surgery. Research demonstrates that PRP positively influences human osteoblast-like cells, facilitating both bone regeneration and wound healing [9]. In endodontic surgery, APCs serve as scaffolds to aid in the reconstruction of the periodontium and bone surrounding the tooth root, thereby promoting healing in the periapical region post-surgery [10,11,12,13]. Studies have indicated notable enhancements in healing rates with the use of APCs [10,11]. Additionally, platelet-rich fibrin has been associated with improved clinical outcomes, such as reduced probing depth [13].

Several prior reviews have investigated the impact of various regenerative techniques in endodontic surgery, including GTR, grafting materials, and APCs. Although some studies have indicated substantial advancements in surgical outcomes through the application of these methods [4,14,15,16], other research has failed to demonstrate any discernible improvements, especially in cases involving smaller apical lesions [17,18,19]. While existing studies offer insights, current evidence is inconclusive regarding the consistent effectiveness of regenerative techniques and materials in improving outcomes. This systematic review and meta-analysis aimed to investigate the impact of bone grafts, membranes, and platelet concentrates on clinical outcomes following endodontic periapical surgery. The primary objective was to test the null hypothesis that there is no significant difference in clinical outcomes when utilizing these regenerative materials.

## 2. Method

The methodology and results of our study were reported in accordance with the Preferred Reporting Items for Systematic Reviews and Meta-Analyses (PRISMA) statement (Supplementary Table S1). This systematic review was prospectively registered in the PROSPERO database (CRD42024528101). The eligibility criteria were defined as follows:

- Population: Individuals with permanent teeth undergoing endodontic surgery.
- Interventions: Endodontic surgery with placement of bone grafts, membranes, or platelet concentrates.
- Comparisons: Endodontic surgery without the placement of bone grafts, membranes, or platelet concentrates.
- Outcomes: Improved clinical and radiographic outcomes one-year post-surgery.
- Study Design: RCTs and prospective clinical trials.

The inclusion criteria encompassed randomized clinical trials or prospective clinical trials; studies focusing on the treatment of permanent teeth undergoing endodontic surgery; studies assessing clinical and radiographic success one-year post-surgery; studies with an intervention group employing various bone grafts, membranes, or platelet concentrates; studies featuring a control group with no utilization of various grafts, platelet concentrates, or membrane; studies conducting a minimum 1-year follow-up evaluation after endodontic surgery; and studies presenting data in a standardized format for proper comparative analysis. The following study types were excluded: retrospective studies, case-control studies, case series, case reports, animal and in vitro studies. Studies were also excluded if they did not meet the inclusion criteria, had unavailable full text, or lacked sufficient methodological detail.

### 2.1. Search Methods for the Identification of Studies

The search terms were selected by three independent reviewers in consultation with librarians. These terms, or their equivalent Medical Subject Headings (MeSH) terms, were utilized to conduct a thorough search across three primary electronic databases: MEDLINE (PubMed), Web of Science, and Embase, spanning from their inception until March 2024 (Supplemental Table S2). To ensure inclusivity, gray literature was incorporated alongside peer-reviewed sources. Google Scholar and ProQuest Dissertations and Theses were employed to identify relevant gray literature. Furthermore, a manual search of references from key articles and book chapters was conducted. The objective was to identify relevant English-language studies meeting the inclusion criteria, accessible through the University of California San Francisco’s libraries. To ensure accurate representation of the results, all located articles were retrieved and imported into EndNote X9 (Thomson Reuters, New York, NY, USA) for systematic management. Duplicate entries were subsequently eliminated to generate a concise reference list.

### 2.2. Screening and Data Extraction

Two reviewers (N.B. and M.R.) conducted independent reviews and selected trials based on initial title/abstract screening. Subsequently, they thoroughly examined the full texts of the identified articles. To ensure consistency between reviewers, a joint evaluation of the first 20 articles was conducted. Any disagreements were resolved through consultation with a third reviewer (M.S.). The criteria for exclusion were then established and applied consistently throughout the review process. The reasons for exclusion at the full-text stage were recorded and presented in Supplemental Table S2.

The following information was extracted from each study and entered into an Excel spreadsheet (Microsoft, Redmond, WA, USA): the year of publication, the number of subjects within each treatment group, the type of treatments received, material types, the duration of the follow-up visits, details of the reported outcomes, including the assessment methods. Two reviewers (N.B. and M.S.) independently verified the accuracy of data extraction.

### 2.3. Risk of Bias Assessment

Two authors (N.B. and M.S.) independently evaluated the risk of bias using the Cochrane Collaboration’s tool. The assessment considered reported information across seven domains for each study, including random sequence generation, allocation concealment, blinding of participants and personnel, blinding of outcomes assessment, incomplete outcome data, selective reporting, and publication bias [20,21].

An allocation of ’low risk’ was assigned when all domains were assessed as having low-bias risk. An allocation of ‘some concerns’ was made if any domain was deemed to have unclear bias risk, and an allocation of ’high risk’ occurred if any domain was assessed as having high-bias risk. Any study with an unclear risk of bias in the blinding of outcome assessors was categorized as high-bias risk.

### 2.4. Quality of Evidence

The Grading of Recommendations, Assessment, Development, and Evaluation (GRADEpro GDT: GRADEpro Guideline Development Tool; McMaster University, Hamilton, ON, Canada) served as the tool for impartially assessing the quality of outcome analyses [22]. Two reviewers (N.B. and M.R.) individually evaluated the quality of evidence, considering five domains: risk of bias, inconsistency of results, indirectness of evidence, imprecision, and publication bias. Any disagreements were resolved through consensus by consulting the third reviewer (M.S.).

### 2.5. Quantitative Analyses

For each intervention, we compiled data on the number of teeth affected by the event (outcome) and the total number of participants in both the intervention and control groups. Meta-analyses were conducted for studies with similar comparisons and reporting identical outcome measures. We utilized a random-effects model for meta-analysis, specifically the DerSimonian and Laird method, to estimate the overall effect while considering the potential heterogeneity between studies. The data were analyzed using Stata software version 14 [23]. To estimate the odds ratio (OR) of the effect of a graft, membrane, or concentrated growth factor on the success rate of root canal surgery using a two-by-two table, the number of individuals with successful and unsuccessful treatment was extracted and categorized by intervention and control groups from each of the primary studies. In interpreting odds ratios (OR), we assess statistical significance by checking if the confidence interval (CI) around the OR does not contain the number 1. If 1 falls outside the CI, it suggests a statistically significant difference between the intervention and control groups. To assess the consistency of findings across the included studies, we evaluated heterogeneity using the I-squared statistic and the Q statistic. Additionally, we employed a funnel plot and Egger’s test to investigate the possibility of publication bias, which can occur when studies with non-significant or negative results are less likely to be published. Sensitivity analysis was also performed to examine the impact of each primary study on the overall estimate.

Data were stratified into subgroups based on the type of regenerative material used during treatment. The subgroups consisted of treatment with bone graft only, treatment with bone graft and membrane, treatment with membrane only, treatment with concentrated growth factor only, and treatment with concentrated growth factor and bone graft. Sensitivity analyses were conducted to assess the impact of studies with a high risk of bias on the results.

## 3. Results

### 3.1. Search Results

**Figure 1:**
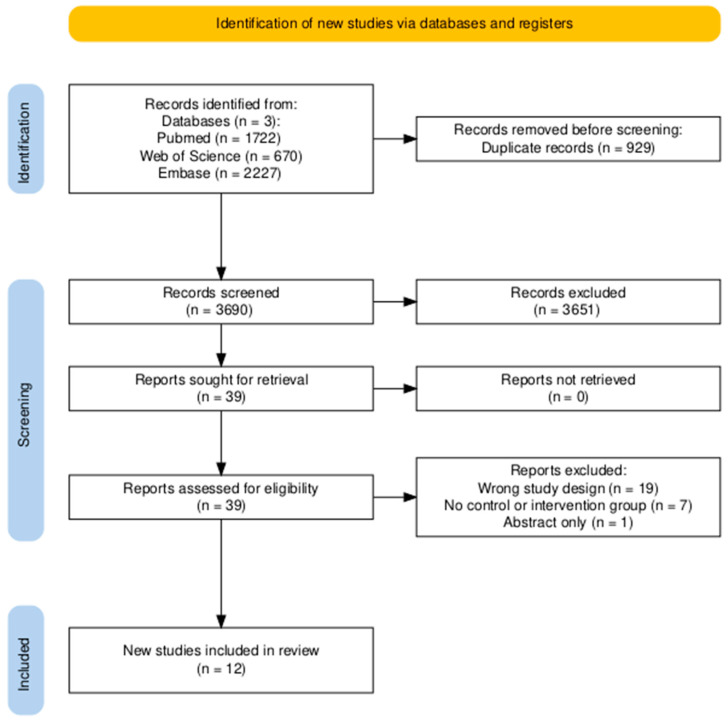
A flow diagram was created to illustrate the research methodology, adhering to the guidelines of the PRISMA statement.

The study selection process is summarized in Figure 1. From the initial search of 4619 records, 929 duplicates and an additional 3651 records were removed by title and abstract screening.

A total of 39 records were screened in full text. Twelve RCTs and prospective clinical trials met our inclusion criteria (Table 1). 27 articles were excluded due to wrong study design, no control/intervention group, or because only the abstract was published (Supplemental Table S3).

### 3.2. Study Characteristics

#### 3.2.1. Population

The 12 studies included permanent teeth that had undergone surgical endodontic retreatment through apicoectomy [2,11,12,24,25,26,27,28,29,30,31,32]. The minimum follow-up time for each study was 1 year. Success was defined as patients who demonstrated clinical success and completed radiographic healing.

#### 3.2.2. Intervention

The interventions included placement of a bone graft only, bone graft with a membrane, membrane only, concentrated growth factor, or concentrated growth factor with a bone graft [2,11,12,24,25,26,27,28,29,30,31,32].

#### 3.2.3. Outcome Assessment

Post-operative outcomes in each study were evaluated clinically and radiographically. Treatment success was determined by the superior performance of regenerative techniques compared to control groups. All tested materials and combinations exhibited improved healing outcomes relative to the control group. Furthermore, healed lesions treated with regenerative techniques demonstrated bone density more closely approximating that of natural bone compared to the control group.

### 3.3. Risk of Bias

**Figure 2:**
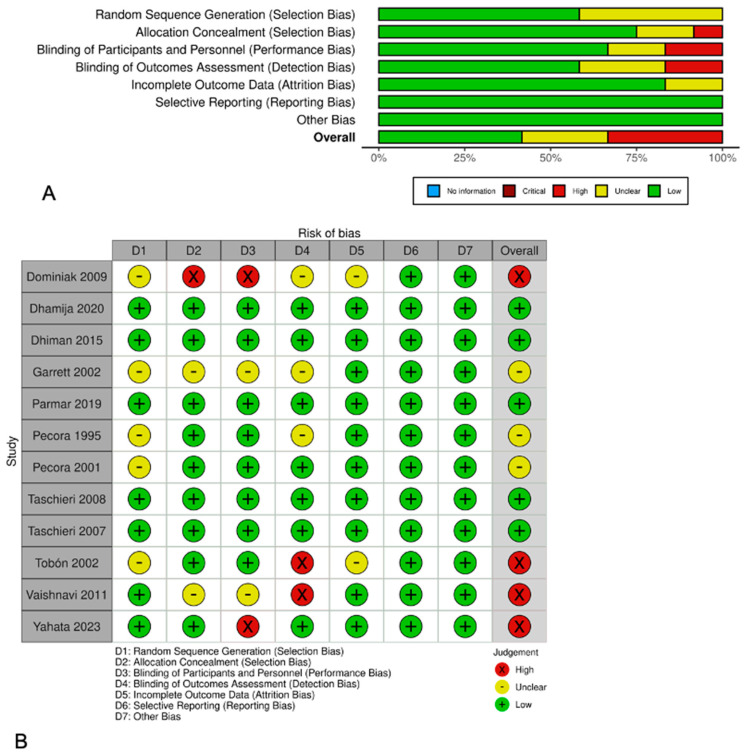
(**A**) An overview of the authors’ assessments regarding each domain of bias, expressed as percentages across the studies included in the analysis. (**B**) A synopsis of the bias risk observed in the studies included [2,11,12,24,25,26,27,28,29,30,31,32].

The assessment of each risk of bias domain in individual studies is depicted in Figure 2A,B [21]. Five studies were considered low risk [2,11,12,26,29]. Three studies had some concerns regarding random sequence generation, allocation concealment, blinding of participants and personnel, and blinding of outcomes assessment [25,27,28], and four studies [24,30,31,32] had a high risk of bias, primarily in random sequence generation, allocation concealment, and blinding of participants and outcome assessment.

## 4. Data Synthesis

### 4.1. Primary Analysis

**Figure 3:**
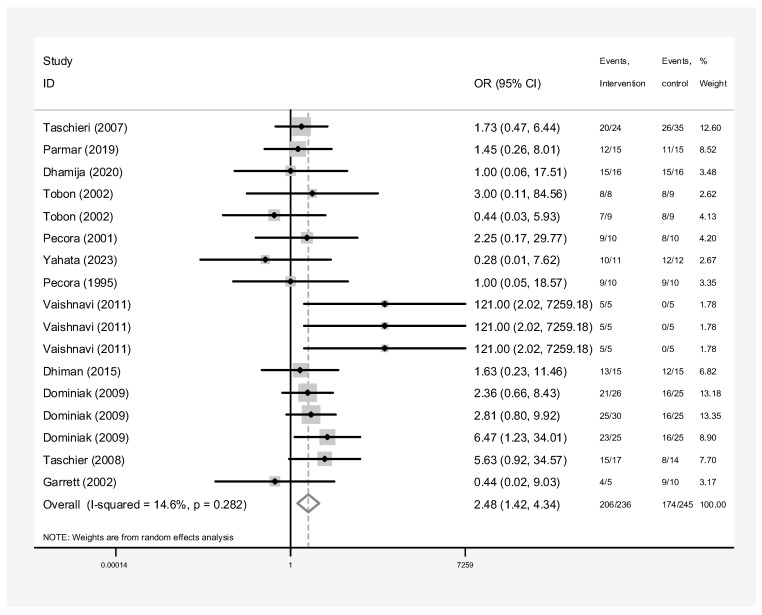
Pooled success rate of any graft, membrane, or concentrated growth factor in endodontic surgery [2,11,12,24,25,26,27,28,29,30,31,32].

**Figure 4:**
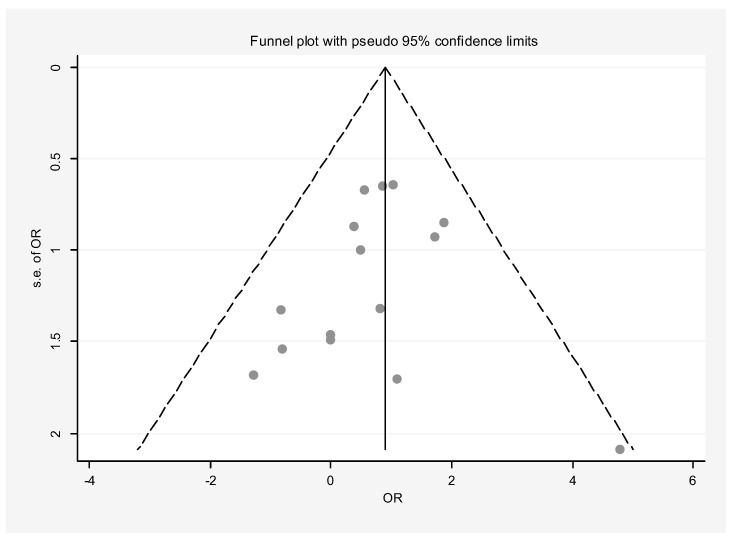
Funnel plot of publication bias showing no publication bias amongst the primary studies.

**Figure 5:**
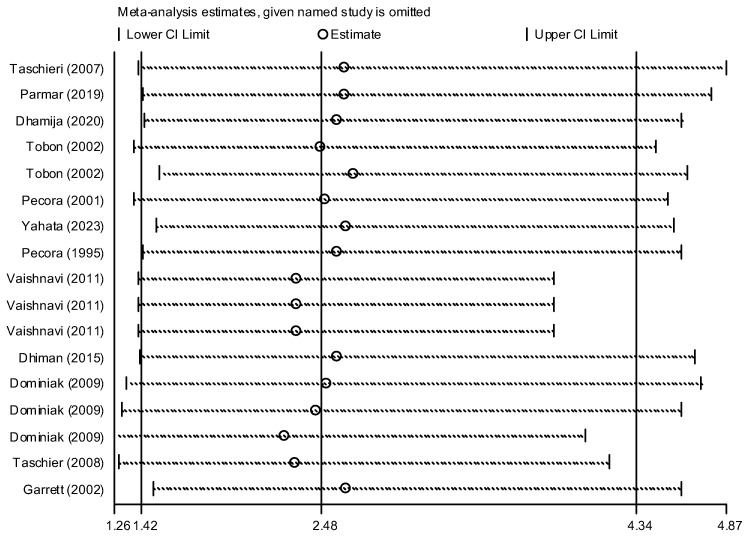
Sensitivity analysis chart to check the effect of each study on the overall estimate [2,11,12,24,25,26,27,28,29,30,31,32].

Figure 3 presents the outcomes of surgery involving any graft, membrane, or concentrated growth factor. The intervention group comprised 236 samples, while the control group had 245 samples. The overall success rate of the surgeries involving grafts, membranes, or growth factors was significantly higher than the control group. Pooling data from these 12 studies, the success rate of root canal surgery with a graft, membrane, or concentrated growth factor was calculated to be 2.48 (OR: 2.48, 95% CI: 1.42–4.34) compared to the control group (Figure 3). Heterogeneity analysis suggests that the variation among the primary study results is not statistically significant (I-squared: 14.6%, Q: 18.74, *p*-value: 0.282). Additionally, both the funnel plot (Figure 4) and Egger’s test (β: 0.53, *p*-value: 0.459) suggest no publication bias. Sensitivity analysis indicates that each primary study’s effect on the overall estimate is consistent (Figure 5). Meta-regression analysis also confirmed that intervention type was not a significant source of heterogeneity (*p* = 0.414). In other words, the effect size did not differ considerably between studies based on the type of intervention used (β = −6.47).

### 4.2. Subgroup Analysis

We conducted a meta-analysis of several treatment modalities for bone regeneration in patients undergoing apical surgery, with a 12-month follow-up period. The treatments included bone graft only, bone graft with membrane, membrane only, concentrated growth factor, and at least one intervention.

**Figure 6:**
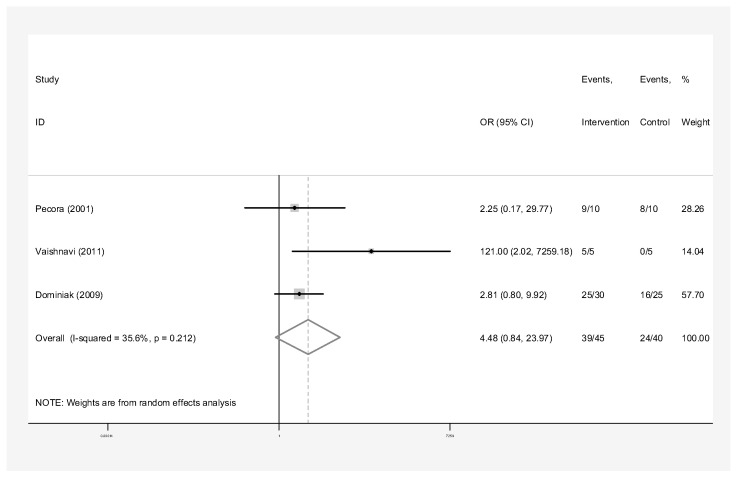
Treatment with bone graft only. Forrest plot diagram of the odds ratio of the effect of bone graft only on the success rate of endodontic surgery compared to the control group, separately for each of the primary evidence and the overall estimate with a 95% confidence interval [24,28,31].

**Figure 7:**
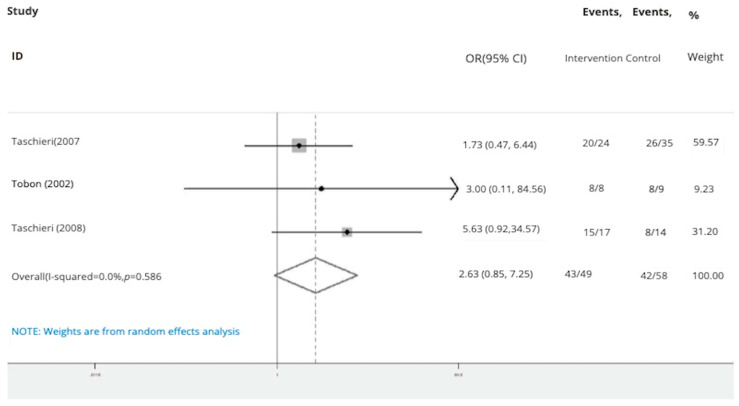
Treatment with bone graft and membrane. Forrest plot diagram of the odds ratio of the effect of bone graft with membrane on the success rate of endodontic surgery compared to the control group, separately for each of the primary evidence and the overall estimate with a 95% confidence interval [2,29,30].

**Figure 8:**
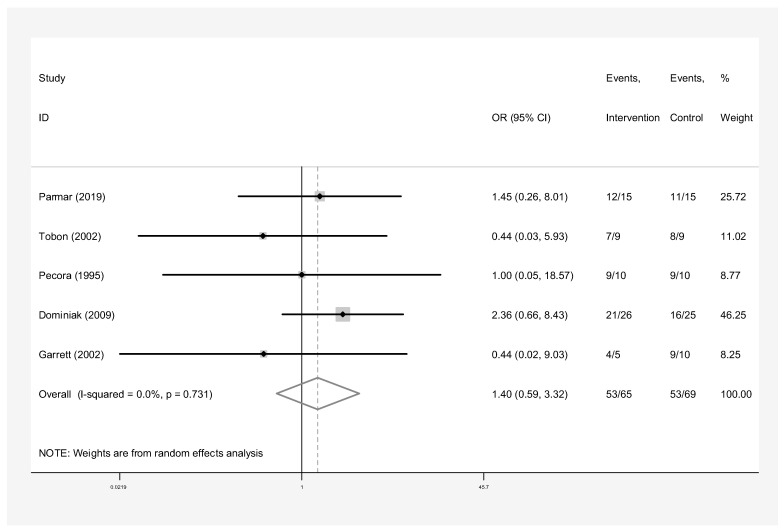
Treatment with membrane only. Forrest plot diagram of the odds ratio of the effect of membrane only on the success rate of endodontic surgery compared to the control group, separately for each of the primary evidence and the overall estimate with a 95% confidence interval [24,25,26,27,30].

**Figure 9:**
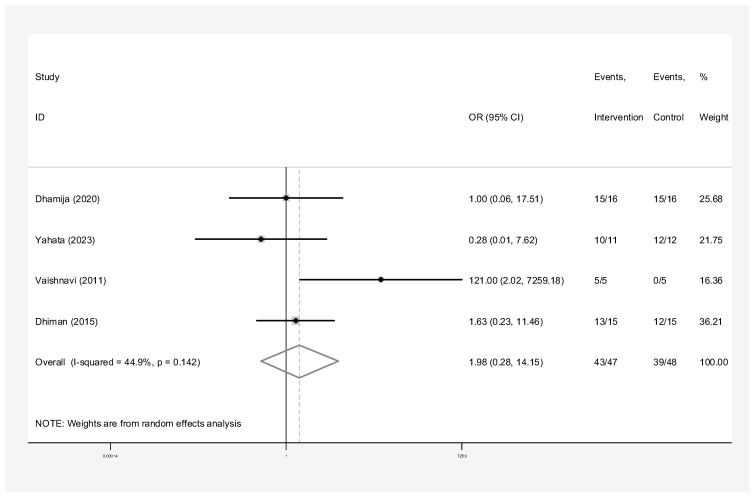
Treatment with concentrated growth factor. Forrest plot diagram of the odds ratio of the effect of concentrated growth factor only on the success rate of endodontic surgery compared to the control group, separately for each of the primary evidence and the overall estimate with a 95% confidence interval [11,12,31,32].

**Figure 10:**
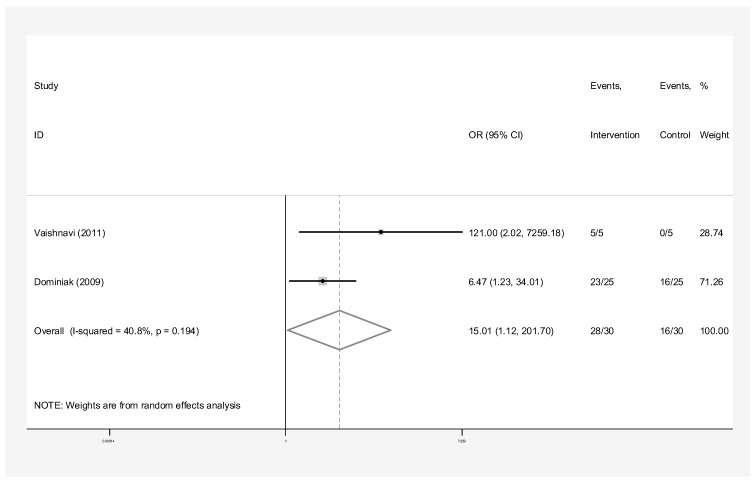
Treatment with concentrated growth factor with bone graft. Forrest plot diagram of the odds ratio of the effect of concentrated growth factor with bone graft on the success rate of endodontic surgery compared to the control group, separately for each of the primary evidence and the overall estimate with a 95% confidence interval [24,31].

The subgroup analyses by the type of material used for pooled success rates were as follows:

- Bone graft only: Intervention group n = 45 and control group n = 40 in three studies [24,28,31]. The success rate of endodontic surgery with graft was estimated to be 4.48 (OR: 4.48, 95% CI: 0.84–23.97) compared to the control group (Figure 6). The statistical heterogeneity for this group was moderately significant (I-squared: 35.6%, Q: 3.10, *p*-value: 0.212). However, this difference was not statistically significant.
- Bone graft with membrane: In three studies (n = 49 intervention, n = 58 control) [2,29,30], the success rate of endodontic surgery with graft and membrane was estimated to be 2.63 times higher (OR: 2.63, 95% CI: 0.95–7.25) compared to the control group (Figure 7). Heterogeneity indices indicate that the heterogeneity between the results of the primary studies is very low (I-squared: 0%, Q: 1.07, *p*-value: 0.586). This difference was not statistically significant.
- Membrane only: Intervention group n = 65 and control group n = 69 in five studies [24,25,26,27,30]. The success rate of endodontic surgery with membrane only was estimated to be 1.40 (OR: 1.40, 95% CI: 0.59–3.32) compared to the control group (Figure 8). Heterogeneity indices indicate that the heterogeneity between the results of the primary studies was very low (I-squared: 0%, Q: 2.03, *p*-value: 0.731). This difference was not statistically significant.
- Concentrated Growth Factor Only: Intervention group n = 47 and control group n = 48 in four studies [11,12,31,32]. The success rate of endodontic surgery with CGF only was estimated to be 1.98 (OR: 1.98, 95% CI: 0.28–14.15) compared to the control group (Figure 9). The statistical heterogeneity was moderate (I^2^: 44.9%, Q: 5.45, *p*-value: 0.142). However, this difference was not statistically significant.
- Concentrated Growth Factor with Bone Graft: Intervention group n = 30 and control group n = 30 in two studies [24,31]. The success rate of endodontic surgery with CGF and bone graft was estimated to be 15.01 (OR: 15.01, 95% CI: 1.12–271.70) compared to the control group (Figure 10). The statistical heterogeneity was moderate (I-squared: 40.8%, Q: 1.69, *p*-value: 0.194). However, this difference was not statistically significant.

## 5. Discussion

The primary objective of this systematic review was to assess the impact of bone grafts, membranes, and platelet concentrates on outcomes in endodontic periapical surgery, utilizing a rigorous evidence-based methodology. The findings of this review did support the null hypothesis. This suggests that the use of concentrated growth factors in conjunction with bone grafts significantly improves the success of bone regeneration procedures.

The comprehensive analysis of all twelve studies investigating the impact of grafts, membranes, or concentrated growth factors on the success rate of endodontic surgery compared to a placebo control group provides robust insights. With a total of 236 samples in the intervention group and 245 in the control group, the results demonstrate a statistically significant improvement in the success rate of surgeries using a regenerative material. Analyzing data from multiple studies, the odds of success were 2.48 (95% CI: 1.42–4.34) times higher for surgeries using grafts compared to no grafts (control group). Despite not reaching statistical significance, regenerative materials in periapical surgery resulted in an improvement in success rates, although more studies are needed for confirmation.

In the subgroup of studies focusing on treatment with bone graft and membrane, the meta-analysis conducted within this research did not reveal a significant improvement in success rates of apical surgeries compared to interventions without graft or membrane. An odds ratio of 2.63 with a wide confidence interval suggests this combination may have minimal effect on improving bone regeneration outcomes [2,29,30]. In contrast to the findings of this review, Tsesis et al. [33] determined that membrane type played a significant role in the success of GTR procedures compared to control cases. Specifically, GTR techniques were observed to have a positive impact on the success of surgical endodontic procedures, particularly in instances involving large periapical lesions and through-and-through lesions. Additionally, the utilization of a resorbable membrane was associated with more favorable outcomes compared to solely using a non-resorbable membrane or graft.

The subgroup analysis focusing solely on membrane treatment revealed no significant increase in success rate compared to interventions without membrane or graft. The odds ratio of 1.40 suggests a negligible difference in outcomes, and the confidence interval encompasses values indicative of no effect. These findings suggest that membrane use alone may not significantly enhance bone regeneration success [24,25,26,27,30]. This observation aligns with a meta-analysis by Deng et al. [4], which concluded that neither resorbable nor non-resorbable membranes demonstrated superior healing rates compared to controls. Additionally, Flynn et al. [15] determined that resorbable membranes used alone did not exhibit a significant enhancement in the healing process of apical lesions following surgery. Flynn et al. [15] also suggested that the type of membrane did not affect healing outcomes. Furthermore, ref. [30] also documented reduced success rates with e-PTFE membranes (77.78%) compared to control groups (88.89%). Non-absorbable alternatives were found to be linked with lower success rates compared to resorbable membranes. In contrast, previous studies, such as the meta-analysis conducted by Tsesis et al. [33], suggested positive outcomes associated with the use of resorbable membranes. Similarly, Liu et al. [16] found evidence suggesting that resorbable membranes used alone could potentially expedite the healing process.

The meta-analysis of studies focusing solely on bone graft treatment over a 12-month follow-up period did not demonstrate a significant increase in success rate compared to interventions without bone graft. The odds ratio of 4.48 suggests the success rate of periapical surgery with graft is estimated to be higher than that of the control group. However, the wide confidence interval indicates considerable uncertainty in the precision of this estimate. The small number of studies is what limits the reliability of this finding [24,28,31]. While numerous studies investigate the effects of GTR, which involves combining a bone graft with a membrane to achieve regeneration, there is a limited amount of research examining the sole impact of bone grafting without any membrane. Flynn et al. [15] similarly found that the effect of bone grafting without a membrane was shown to increase the likelihood of success, but the improvement was not significant. Currently, it can be inferred that the advantages of utilizing a grafting material without a membrane remain uncertain.

The evaluation of the outcomes of endodontic surgery using concentrated growth factor (CGF) alone and in combination with bone graft provides insightful comparisons. In the analysis of CGF only, the success rate was estimated with an odds ratio of 1.98. Conversely, when CGF was combined with bone grafts, the success rate showed a markedly higher odds ratio of 15.01. Despite the significant increase in odds ratio, the wide confidence interval reflects substantial uncertainty, likely due to the smaller sample size. These findings suggest that while CGF alone shows potential, the combination with bone grafts may significantly enhance the success rate of endodontic surgeries, though further studies with larger sample sizes are warranted to validate these preliminary results [11,12,31,32]. Similarly, Meschi et al. [34] observed that autologous platelet concentrates (APCs) offer positive effects in various other endodontic procedures. According to Meschi et al. [34], APCs expedite postoperative bone healing, enhance postoperative quality of life for patients, facilitate additional root development, and assist in maintaining or restoring pulp vitality. Liu et al. [16] similarly discovered that the utilization of autologous platelet concentrates (APCs) led to improved healing outcomes following endodontic surgery, although they did not observe a statistically significant difference.

The recent systematic review and meta-analysis by Flynn et al. [15] presented similar findings, suggesting that regenerative techniques involving bone grafting, with or without barrier membrane placement, notably improved the outcomes of endodontic apical surgery. However, this study acknowledges a potential limitation stemming from the small sample size of the included studies, which may constrain the statistical power, even when pooling the meta-analysis results. Despite this, Flynn et al. [15] found that subgroup analysis of individual bone grafts, membranes, or their combination did not yield any significant results, aligning with the findings of this study. Flynn et al. [15] also found that the specific type of membrane or the combination of graft and membrane used did not significantly affect outcomes in periapical surgery. Building on this work, our study aims to further explore the impact of regenerative materials by incorporating platelet concentrates into the analysis. This will help us understand the potential benefits of platelet concentrates in apical surgery, alongside the findings of Flynn et al. [15] regarding the necessity of other regenerative materials.

While our study possesses strengths, it also bears limitations. The pooled analyses were derived from a relatively small cohort, primarily focusing on comparing various materials, thereby impacting the domain of ‘indirectness’ in quality assessment. Like any meta-analysis, subtle yet significant differences between interventions and baseline characteristics may exist, potentially leading to a variance between estimated and actual outcomes. Furthermore, the certainty of our findings concerning intervention safety is constrained. One notable limitation of our study pertains to sample size. Our pooled analyses were drawn from a relatively modest number of participants, potentially constraining the generalizability of our findings to broader populations.

This study demonstrates several strengths. To minimize bias and ensure studies were relevant and comparable for analysis, this systematic review employed strict inclusion criteria and a comprehensive search strategy. We focused on studies with rigorous methodologies that directly addressed the research question. This methodological approach strengthens the consistency between studies and lays the groundwork for reliable meta-analyses. By exclusively focusing on randomized controlled trials (RCTs) and prospective cohorts with a control group, this review ensures a high level of internal validity and enables causal inferences regarding intervention effectiveness. Additionally, this study conducted a novel subgroup analysis that investigated the effects of each regenerative material both individually and in combination, providing a more nuanced understanding of their efficacy compared to previous reviews. The included studies exhibited a high level of homogeneity (I-squared: 14.6%, Q: 18.74, *p*-value: 0.282), bolstering confidence in the pooled estimates and the validity of the meta-analysis results.

Based on the comprehensive analysis conducted in this systematic review, several avenues for future research emerge to deepen our understanding of interventions in bone regeneration following endodontic surgeries. Given the variability observed in the literature regarding the efficacy of different regenerative materials, future studies could delve into elucidating the mechanisms underlying these variations. Exploring factors such as lesion size, lesion type, and membrane characteristics may help discern the optimal approach for enhancing bone regeneration outcomes. Additionally, investigating the long-term effects of interventions beyond the 12-month follow-up period covered in many studies could provide valuable insights into the sustainability of treatment effects over time. Moreover, addressing the limitations identified in this study, particularly regarding the small sample size, will be essential for enhancing the robustness and generalizability of future research findings. Given the limitations of this review, including small sample sizes, heterogeneity in study methodologies and designs, limited variability in patient characteristics and study populations, a small number of clinical cases per study, and a short follow-up period (12 months), larger-scale studies are imperative to definitively determine the true impact of guided bone regeneration and the use of regenerative materials on clinical outcome. By addressing these gaps, future studies can contribute to advancing our understanding of optimal interventions for promoting bone regeneration and improving outcomes in endodontic surgeries.

This review provides valuable insights for endodontists in clinical practice, particularly regarding the use of regenerative materials in apical surgeries. Through a focused meta-analysis comparing the efficacy of regenerative materials to conventional surgical techniques, this review aids clinicians in assessing grafting as a viable treatment option for their patients. The analysis revealed statistically significant improvements in success rates with the use of bone grafts, membranes, or platelet concentrates, suggesting that clinicians should consider the use of a regenerative material during surgery. Endodontists are also encouraged to consider various factors such as lesion size, lesion type, and membrane characteristics when selecting treatment modalities, as these factors have been demonstrated to influence outcomes.

## 6. Conclusions

Overall, the findings indicate that utilizing concentrated growth factors with a bone graft significantly improve the success of bone regeneration procedures over a 12-month follow-up period compared to interventions without these components. However, it is important to consider the limitations such as the small number of studies and the need for further research to establish more conclusive evidence regarding the efficacy of these interventions in bone regeneration.

## Figures and Tables

**Table 1:** Characteristics of the included studies.

					Intervention Group			Control Group		
Author, Date	Age Range	Lesion Size/Type	Tooth Type	Regenerative Material	Cases	Success	Failure	Cases	Success	Failure
Dominiak 2009 [24]	9 to 60	Von Arx 1a	Maxillary/Mandibular Anterior/Posterior	Resorbable collagen membranes (BioGide^®^, NJ, USA)	26	21	5	25	16	9
				Xenogenic Bio-Oss collagen graft^®^, NJ USA	30	25	5			
				Xenogenic Bio-Oss collagen^®^ material in combination with platelet-rich plasma (PRP)	25	23	2			
Dhamija 2020 [12]	16 years and older	Through and through	Maxillary/Mandibular Tooth type not specified	Platelet-rich plasma (PRP)	16	15	1	16	15	1
Dhiman 2015 [11]	17 to 47	Not defined	Maxillary/Mandibular Tooth type not specified	Platelet-rich fibrin (PRF)	15	13	2	15	12	3
Garrett 2002 [25]	24 to 67	Von Arx 1a	Tooth type not specified	Bioresorbable polylactic acid membrane	10	9	1	5	4	1
Parmar 2019 [26]	16 years and older	Through and through	Maxillary/Mandibular Anterior/Posterior	Resorbable collagen membrane	15	12	3	15	11	4
Pecora 1995 [27]	27 to 50	Through and through	Tooth type not specified	e-PTFE membrane (Gortex)	10	9	1	10	9	1
Pecora 2001 [28]	47 to 50	Von Arx 1a Through and through	Tooth type not specified	Calcium sulphate graft	10	9	1	10	8	2
Taschieri 2008 [2]	Mean age Women–32 Men 47	Through and through	Maxillary/Mandibular Anterior/Posterior	Anorganic bovine hydroxyapatite graft with resorbable collagen membrane	17	15	2	14	8	6
Taschieri 2007 [29]	Mean age Women–d36 Men 43	Through and through and 4 wall defects	Maxillary/Mandibular Anterior/Posterior	Anorganic bovine hydroxyapatite graft with resorbable collagen membrane	24	20	4	35	26	4
Tobón 2002 [30]	14 to 74	Not defined	Maxillary/Mandibular Anterior/Posterior	Synthetic bioactive resorbable graft of hydroxylapatite (Osteogen) with nonbioabsorbable GoreTex1 augmentation membrane	8	8	0	9	8	1
		Not defined		Nonbioabsorbable GoreTex1 augmentation membrane	9	7	2			
Vaishnavi 2011 [31]	20 to 40	Bony defect had to be confined to the apical area with the bone covering the entire root surface coronally and had an intact lingual cortical plate	Tooth type not specified	Platelet-rich plasma (PRP) and hydroxyapatite	5	5	0	5	0	5
				Platelet-rich plasma (PRP)	5	5	0			
				Replacement with hydroxyapatite	5	5	0			
Yahata 2023 [32]	20 to 70	Lesion size ≥5 mm in diameter on periapical radiography	Maxillary/Mandibular Anterior/Posterior	Concentrated growth factor (CGF)	11	10	1	12	12	0

## Data Availability

All data extracted from included studies and data used for all analyses can be found within the contents of this paper and the included tables and figures.

## Supplementary Materials

The following supporting information can be downloaded at: https://www.mdpi.com/article/10.3390/dj13030091/s1, Table S1: PRISMA guidelines. Table S2: This table shows the databases searched and search terms used. Table S3: List of Excluded Studies with Reasons for Exclusion
