# Supplementary material for: Optimizing Endodontic Surgery: A Systematic Review of Guided Tissue Regeneration, Grafting, and Platelet Concentrates vs. No Intervention
Source: Dent J (Basel). 2025 Feb 20;13(3):91. doi: 10.3390/dj13030091 (PMC11941754; doi:10.3390/dj13030091)
Supplement: Supplementary file 1 [file dentistry-13-00091-s001.zip › dentistry-3331456-supplementary.pdf]

Section and Topic	Item #	Checklist item	Location where item is reported
**TITLE**			
Title	1	Identify the report as a systematic review.	Page 1
**ABSTRACT**			
Abstract	2	See the PRISMA 2020 for Abstracts checklist.	Page 1
**INTRODUCTION**			
Rationale	3	Describe the rationale for the review in the context of existing knowledge.	Pages 3 - 5
Objectives	4	Provide an explicit statement of the objective(s) or question(s) the review addresses.	Page 5
**METHODS**			
Eligibility criteria	5	Specify the inclusion and exclusion criteria for the review and how studies were grouped for the syntheses.	Pages 5 - 6
Information sources	6	Specify all databases, registers, websites, organisations, reference lists and other sources searched or consulted to identify studies. Specify the date when each source was last searched or consulted.	Page 6 and Supplementary Table 1
Search strategy	7	Present the full search strategies for all databases, registers and websites, including any filters and limits used.	Pages 6 - 7
Selection process	8	Specify the methods used to decide whether a study met the inclusion criteria of the review, including how many reviewers screened each record and each report retrieved, whether they worked independently, and if applicable, details of automation tools used in the process.	Pages 6 - 7
Data collection process	9	Specify the methods used to collect data from reports, including how many reviewers collected data from each report, whether they worked independently, any processes for obtaining or confirming data from study investigators, and if applicable, details of automation tools used in the process.	Pages 6 - 7
Data items	10a	List and define all outcomes for which data were sought. Specify whether all results that were compatible with each outcome domain in each study were sought (e.g. for all measures, time points, analyses), and if not, the methods used to decide which results to collect.	Pages 6 - 7

Section and Topic	Item #	Checklist item	Location where item is reported
	10b	List and define all other variables for which data were sought (e.g. participant and intervention characteristics, funding sources). Describe any assumptions made about any missing or unclear information.	Pages 6 - 7
Study risk of bias assessment	11	Specify the methods used to assess risk of bias in the included studies, including details of the tool(s) used, how many reviewers assessed each study and whether they worked independently, and if applicable, details of automation tools used in the process.	Pages 7 – 8
Effect measures	12	Specify for each outcome the effect measure(s) (e.g. risk ratio, mean difference) used in the synthesis or presentation of results.	Pages 8 - 9
Synthesis methods	13a	Describe the processes used to decide which studies were eligible for each synthesis (e.g. tabulating the study intervention characteristics and comparing against the planned groups for each synthesis (item #5)).	Pages 6 -7
	13b	Describe any methods required to prepare the data for presentation or synthesis, such as handling of missing summary statistics, or data conversions.	Pages 6 - 7
	13c	Describe any methods used to tabulate or visually display results of individual studies and syntheses.	Pages 6 -7 and Table 1
	13d	Describe any methods used to synthesize results and provide a rationale for the choice(s). If meta-analysis was performed, describe the model(s), method(s) to identify the presence and extent of statistical heterogeneity, and software package(s) used.	Pages 8 - 9
	13e	Describe any methods used to explore possible causes of heterogeneity among study results (e.g. subgroup analysis, meta-regression).	Pages 8 - 9
	13f	Describe any sensitivity analyses conducted to assess robustness of the synthesized results.	Pages 8 - 9
Reporting bias assessment	14	Describe any methods used to assess risk of bias due to missing results in a synthesis (arising from reporting biases).	Pages 7 - 8
Certainty assessment	15	Describe any methods used to assess certainty (or confidence) in the body of evidence for an outcome.	Pages 8 - 9
**RESULTS**			

Section and Topic	Item #	Checklist item	Location where item is reported
Study selection	16a	Describe the results of the search and selection process, from the number of records identified in the search to the number of studies included in the review, ideally using a flow diagram.	Figure 1
	16b	Cite studies that might appear to meet the inclusion criteria, but which were excluded, and explain why they were excluded.	Supplementary Table 2
Study characteristics	17	Cite each included study and present its characteristics.	Table 1
Risk of bias in studies	18	Present assessments of risk of bias for each included study.	Figure 2
Results of individual studies	19	For all outcomes, present, for each study: (a) summary statistics for each group (where appropriate) and (b) an effect estimate and its precision (e.g. confidence/credible interval), ideally using structured tables or plots.	Figures 3 – 10
Results of syntheses	20a	For each synthesis, briefly summarise the characteristics and risk of bias among contributing studies.	Pages 10 - 11
	20b	Present results of all statistical syntheses conducted. If meta-analysis was done, present for each the summary estimate and its precision (e.g. confidence/credible interval) and measures of statistical heterogeneity. If comparing groups, describe the direction of the effect.	Pages 10 – 13
	20c	Present results of all investigations of possible causes of heterogeneity among study results.	Pages 10 – 13
	20d	Present results of all sensitivity analyses conducted to assess the robustness of the synthesized results.	Pages 10 – 13
Reporting biases	21	Present assessments of risk of bias due to missing results (arising from reporting biases) for each synthesis assessed.	Pages 10 – 11
Certainty of evidence	22	Present assessments of certainty (or confidence) in the body of evidence for each outcome assessed.	Pages 10 – 13
**DISCUSSION**			
Discussion	23a	Provide a general interpretation of the results in the context of other evidence.	Pages 13 – 18

Section and Topic	Item #	Checklist item	Location where item is reported
	23b	Discuss any limitations of the evidence included in the review.	Pages 17 – 18
	23c	Discuss any limitations of the review processes used.	Pages 17 – 18
	23d	Discuss implications of the results for practice, policy, and future research.	Page 18
**OTHER INFORMATION**			
Registration and protocol	24a	Provide registration information for the review, including register name and registration number, or state that the review was not registered.	Abstract
	24b	Indicate where the review protocol can be accessed, or state that a protocol was not prepared.	Abstract
	24c	Describe and explain any amendments to information provided at registration or in the protocol.	Abstract
Support	25	Describe sources of financial or non-financial support for the review, and the role of the funders or sponsors in the review.	Abstract and Page 19
Competing interests	26	Declare any competing interests of review authors.	Page 19
Availability of data, code and other materials	27	Report which of the following are publicly available and where they can be found: template data collection forms; data extracted from included studies; data used for all analyses; analytic code; any other materials used in the review.	Page 19

From: Page MJ, McKenzie JE, Bossuyt PM, Boutron I, Hoffmann TC, Mulrow CD, et al. The PRISMA 2020 statement: an updated guideline for reporting systematic reviews. BMJ 2021;372:n71. doi: 10.1136/bmj.n71

Supplementary Table S1: PRISMA guidelines

Supplementary Table 1: Databases and Search Terms	
**Resource**	**Search terms for individual database, targeted journals, or text books**
Pubmed	"Periapical Abscess/surgery"[Mesh] OR “periapical surgery” OR "apical surgery" OR "Apicoectomy"[Mesh] OR apicoectomy OR “guided tissue regeneration” OR “endodontic surgery” OR “endodontic surgeries”) AND ("Bone Transplantation"[Mesh] OR “bone graft” OR “bone thickness” OR “lesion size” OR “size of lesion” OR "Radiography, Dental"[Mesh] OR radiography OR “tissue graft” OR “tissue grafting” OR "Tissue Transplantation"[Mesh] OR “platelet-rich plasma” OR "Platelet-Rich Plasma"[Mesh] OR “platelet-rich fibrin” OR "Platelet-Rich Fibrin"[Mesh] OR membrane OR "Membranes"[Mesh]) AND ("Treatment Outcome"[Mesh] OR “treatment outcome” OR outcome OR outcomes OR "Prognosis"[Mesh] OR prognosis OR "Outcome Assessment, Health Care"[Mesh] OR “long term effects” OR “short term effects” OR retreatment OR "Retreatment"[Mesh] OR prediction OR predictors
Embase	periapical surgery'/exp OR 'periapical surgery' OR 'apical surgery' OR 'apicoectomy'/exp OR apicoectomy OR 'tissue regeneration'/exp OR 'tissue regeneration' OR 'periodontal tissue regeneration'/exp OR 'periodontal tissue regeneration' OR 'endodontic surgery'/exp OR 'endodontic surgery') AND ('bone graft'/exp OR 'bone graft' OR 'bone thickness'/exp OR 'bone thickness' OR 'radiography'/exp OR radiography OR 'tissue graft'/exp OR 'tissue graft' OR 'tissue grafting' OR (lesion AND size) OR 'platelet-rich plasma'/exp OR 'platelet-rich plasma' OR 'platelet-rich fibrin'/exp OR 'platelet-rich fibrin' OR 'membrane'/exp OR membrane)<br><br>AND ('treatment outcome'/exp OR 'treatment outcome' OR 'outcome'/exp OR 'outcome' OR 'outcomes'/exp OR 'outcomes' OR 'prognosis'/exp OR 'prognosis' OR 'retreatment'/exp OR 'retreatment' OR 'long term effects' OR 'short term effects' OR 'prediction'/exp OR prediction OR 'predictors'/exp OR predictors
Web of Science	"periapical surgery" OR "apical surgery" OR apicoectomy OR “guided tissue regeneration” OR “endodontic surgery”) AND (“bone graft” OR “bone thickness” OR “lesion size” OR “size of lesion” OR radiography OR “tissue graft” OR “tissue grafting” OR “platelet-rich plasma” OR “platelet-rich fibrin” OR membrane) AND (“treatment outcome” OR outcome OR outcomes OR prognosis OR “long term effects” OR “short term effects” OR retreatment OR prediction OR predictors
Journals	Journal of Endodontics, The International Journal of Oral and Maxillofacial Surgery, Oral Surgery, Oral Medicine, Oral Pathology, and Oral Radiology. The International Dental Journal, Quintessence International, Medicina Oral Patología Oral y Cirugia Bucal, Saudi Dental Journal, The Journal of the American Dental Association, The Journal of Oral and Maxillofacial Surgery. The Journal of Physiology and Pharmacology, Australian Endodontic Journal, The Journal of the Formosan Medical Association.
Textbooks	Cohen's Pathways of the Pulp, Ingle's Endodontics, and Essential Endodontology: Prevention and Treatment of Apical Periodontitis.

Supplementary Table S2: This table shows the databases searched and search terms used

Supplementary Table 2: Excluded articles and reasons for exclusion.

Study	Title	Reasons for Exclusion
Azim 2021	The Buffalo study: Outcome and associated predictors in endodontic microsurgery- a cohort study	No control group
Baruwa 2023	Management of Apico-marginal Defects With Endodontic Microsurgery and Guided Tissue Regeneration: A Report of Thirteen Cases.	Case report
Deng 2016	The Effect of Regeneration Techniques on Periapical Surgery With Different Protocols for Different Lesion Types: A Meta-Analysis	Meta analysis
Dietrich 2003	Periapical and periodontal healing after osseous grafting and guided tissue regeneration treatment of apicomarginal defects in periradicular surgery: results after 12 months	No control group
Garg 2023	Application of platelet-rich fibrin and freeze-dried bone allograft following apicoectomy: A comparative assessment of radiographic healing	No control group
Goyal 2011	Comparative evaluation of platelet-rich plasma and guided tissue regeneration membrane in the healing of apicomarginal defects: a clinical study	No control group
Han 2022	Platelet-rich fibrin/anorganic bovine bone mineral complex as grafting materials in endodontic microsurgery with a large lesion size: study protocol for a randomised controlled trial	Abstract only accessible
Hiremath 2014	Use of second-generation platelet concentrate (platelet-rich fibrin) and hydroxyapatite in the management of large periapical inflammatory lesion: a computed tomography scan analysis	Case report
Liu 2021	Impact of different regenerative techniques and materials on the healing outcome of endodontic surgery: a systematic review and meta-analysis	Systematic review and meta analysis
Meschi 2020	Multi-modular bone healing assessment in a randomized controlled clinical trial of root-end surgery with the use of leukocyte- and platelet-rich fibrin and an occlusive membrane	Outcome assessment did not report the number of successful vs. failed cases
Oh 2009	Treatment strategy for guided tissue regeneration in combined endodontic-periodontal lesions: case report and review	Case report
Pantchev 2009	Endodontic surgery with and without inserts of bioactive glass PerioGlas--a clinical and radiographic follow-up	No control group
Sánchez-Torres 2014	Materials and prognostic factors of bone regeneration in periapical surgery: a systematic review	Systematic review
Schliephake 1994	Enhancement of bone ingrowth into a porous hydroxylapatite-matrix using a resorbable polylactic membrane: an experimental pilot study	Animal study
Shivashankar 2013	Combination of platelet rich fibrin, hydroxyapatite and PRF membrane in the management of large inflammatory periapical lesion	Case report
Soto-Peñaloza 2019	Pain and quality of life after endodontic surgery with or without advanced platelet-rich fibrin membrane application: a randomized clinical trial	Study assessed pain/quality of life rather than clinical/radiographic healing
Sumangali 2021	Bone Regenerative Biomaterials in Periapical Surgery: A Systemic Review and Meta-Analysis	Systematic review and meta analysis
Sumangali 2021	Various Assisted Bone Regeneration in Apicectomy Defects Systematic Review and Meta Analysis	Systematic review and meta analysis
Sbricoli 2020	Selection of Collagen Membranes for Bone Regeneration: A Literature Review	Literature review
Taschieri 2012	Treatment of through-and-through bone lesion using autologous growth factors and xenogeneic bone graft: a case report	Case report
Taschieri 2011	Effect of guided tissue regeneration on the outcome of surgical endodontic treatment of through-and-through lesions: a retrospective study at 4-year follow-up	Retrospective study
Ustaoğlu 2020	Comparison of GTR, T-PRF and open-flap debridement in the treatment of intrabony defects with endo-perio lesions: a randomized controlled trial	Follow-up time was only 9 months
Uppada 2017	Combination of hydroxyapatite, platelet rich fibrin and amnion membrane as a novel therapeutic option in regenerative periapical endodontic surgery: Case series	Case series
Von Arx 2019	A 10-year Follow-up Study of 119 Teeth Treated with Apical Surgery and Root-end Filling with Mineral Trioxide Aggregate	No intervention group
Von Arx 2007	Clinical and radiographic assessment of various predictors for healing outcome 1 year after periapical surgery	No intervention group
Wang 2023	The effects of different regenerative technologies and materials on wound healing after surgical endodontic therapy: A meta-analysis	Meta analysis
Yoshikawa 2002	Guided bone regeneration (GBR) using membranes and calcium sulphate after apicectomy: a comparative histomorphometrical study	Animal study

Supplementary Table S3: List of Excluded Studies with Reasons for Exclusion
