# Supplementary material for: Optimizing Endodontic Surgery: A Systematic Review of Guided Tissue Regeneration, Grafting, and Platelet Concentrates vs. No Intervention
Source: Dent J (Basel). 2025 Feb 20;13(3):91. doi: 10.3390/dj13030091 (PMC11941754; doi:10.3390/dj13030091)
Supplement: Supplementary file 1 [file Cover Letter_.pdf › Cover Letter_.pdf]

December 31, 2024

We would like to submit our manuscript entitled "Optimizing Endodontic Microsurgery: A Systematic Review of Guided Tissue Regeneration, Grafting and Platelet Concentrates vs. No Intervention"

In this manuscript, we aim to evaluate existing randomized controlled trials (RCTs) and prospective clinical trials to determine the efficacy of bone grafts, membranes, or platelet concentrates on outcomes in endodontic periapical surgery.

We are confident that our findings will be of interest to the readership of the Journal of Endodontics.

This is an original manuscript that has been submitted solely to the Dentistry Journal and that is not concurrently under consideration for publication in another journal. Furthermore, all authors have been involved in the work leading to the publication of the paper and have read and approved this final version. We have no conflicts of interest to disclose. We affirm that we have no financial affiliation (e.g., employment, direct payment, stock holdings, retainers, consultantships, patent licensing arrangements, or honoraria) or involvement with any commercial organization with a direct financial interest in the subject.

Thank you for considering our manuscript for publication in the Dentistry Journals. We look forward to the opportunity to contribute to the scientific literature in this field. Please address correspondence concerning this submission to mike.sabeti@ucsf.edu

Thank you for your consideration of this manuscript.
